# Health-ID: A Blockchain-Based Decentralized Identity Management for Remote Healthcare

**DOI:** 10.3390/healthcare9060712

**Published:** 2021-06-10

**Authors:** Ibrahim Tariq Javed, Fares Alharbi, Badr Bellaj, Tiziana Margaria, Noel Crespi, Kashif Naseer Qureshi

**Affiliations:** 1 Lero-Science Foundation Ireland Research Centre for Software, University of Limerick, V94 T9PX Limerick, Ireland; tiziana.margaria@ul.ie; 2 Computer Science Department, Shaqra University, Shaqra 15526, Saudi Arabia; faalhrbi@su.edu.sa; 3 Institut Mines-Télécom, Télécom SudParis, CEDEX, 91011 Evry, France; badr.bellaj@telecom-sudparis.eu (B.B.); noel.crespi@mines-telecom.fr (N.C.); 4 The Health Research Institute (HRI), University of Limerick, V94 T9PX Limerick, Ireland; 5 Department of Computer Science, Bahria University, Islamabad 44000, Pakistan; knaseer.buic@bahria.edu.pk

**Keywords:** digital identity, decentralized identity, identity management, healthcare, blockchain, smart contract, Ethereum

## Abstract

COVID-19 has made eHealth an imperative. The pandemic has been a true catalyst for remote eHealth solutions such as teleHealth. Telehealth facilitates care, diagnoses, and treatment remotely, making them more efficient, accessible, and economical. However, they have a centralized identity management system that restricts the interoperability of patient and healthcare provider identification. Thus, creating silos of users that are unable to authenticate themselves beyond their eHealth application’s domain. Furthermore, the consumers of remote eHealth applications are forced to trust their service providers completely. They cannot check whether their eHealth service providers adhere to the regulations to ensure the security and privacy of their identity information. Therefore, we present a blockchain-based decentralized identity management system that allows patients and healthcare providers to identify and authenticate themselves transparently and securely across different eHealth domains. Patients and healthcare providers are uniquely identified by their health identifiers (healthIDs). The identity attributes are attested by a healthcare regulator, indexed on the blockchain, and stored by the identity owner. We implemented smart contracts on an Ethereum consortium blockchain to facilities identification and authentication procedures. We further analyze the performance using different metrics, including transaction gas cost, transaction per second, number of blocks lost, and block propagation time. Parameters including block-time, gas-limit, and sealers are adjusted to achieve the optimal performance of our consortium blockchain.

## 1. Introduction

Since SARS-CoV-2 (COVID-19) emerged, the demand for eHealth has gone viral. The novel coronavirus has swept across communities forcing a new normal that requires social distancing. Governments strongly suggest enforcing medical distancing to minimize physical contact between patients and healthcare providers. As a result, hospitals and other healthcare organizations have rapidly adopted digital alternatives to deliver healthcare services. Telehealth applications facilitate clinical benefits for patients such as consultation, diagnoses, treatment, and prevention from a distance overcoming the geographical barrier. They can also support non-clinical services for healthcare providers, such as training, meetings, and education [1]. Furthermore, eHealth applications also provide real-time health monitoring using various devices and sensors [2]. In 2020, remote healthcare applications shifted from a previously slow adoption rate to a record pace of uptake. The searches for online consultations have sky rocked by 350%. Whereas in 2021, the global eHealth market is expected to witness a 37.1% increase [3]. It is further predicted to rise to USD 310.09 billion by 2027, according to Data Bridge Market Research [4].

Healthcare organizations’ rapid transition to digital—where medical records and online services are the norms—has created new challenges in securing access to sensitive patient data and clinical applications. This, combined with evolving compliance regulations, drives a need for technologies that enhance security while maintaining a superior level of healthcare service and enabling healthcare professionals to securely and seamlessly access patient information and applications at all times in compliance with regulatory requirements. However, meeting regulatory demands and demonstrating compliance can be challenging with centralized legacy identity management (IdM) solutions. Failure to comply can result in substantial fines and reputation damage. Furthermore, the proliferation of healthcare organization data breaches [5], which put lives at risk, make centralized legacy identity management (IdM) solutions less desirable.

The centralized IdM creates silos of users restricting inter-operable identification between different applications [6]. For each application, the consumer has to identify and authenticate themselves separately. A user identified in one domain cannot verify itself to a user present in another domain. Moreover, the web-based IdM allows users to create self-asserted profiles without performing any identity proofing. Therefore, identity theft is becoming common on web applications to commit scams and frauds [7,8]. Furthermore, centralized IdM has several weaknesses in ensuring data security and privacy [9]. In a centralized IdM, the identity owner must completely trust their service provider, thus having no or limited control over their identity. Users are unable to control the type of data collected and shared during the identification and authentication process. There remains no way for a consumer to check whether the security and privacy regulations are being followed by their service provider [10]. Moreover, centralized IdM remains more prone to hacking and data breaches, which may lead to susceptible data disclosure for eHealth applications [11]. In centralized IdM, the data of users are stored, updated, and managed through centralized databases. Centralized databases are high-value targets for different security attacks, thus remain more prone to data manipulation or theft. Centralized servers also introduce a single point of failure and are venerable to Denial-of-Service attacks. If the centralized system is compromised, it may make the network completely useless. Thus, a centralized approach always requires an adequate security system with protective, detective, and corrective measures to protect against different security threats. Therefore, decentralized methods are being proposed in  [12,13,14] to ensure data security and privacy.

For remote eHealth applications, it remains essential to provide a decentralized IdM. What is required is a facility that empowers users to manage their identities independently from their eHealth provider. Blockchain technology allows distributed storage of records with cryptography protection to ensure security [15]. It facilitates decentralization and stores time-stamped data in an immutable, auditable, and secure manner. These features can be used to facilitate the owner-driven identity. In the survey in Reference [16], a blockchain-based IdM is suggested for eHealth consumers to control their identity fully. There are several examples of blockchain-based IdM, including ShoCard [17], uPort [18], Sovrin [19], and Blockstack [20]. However, these solutions are not directly applicable to telehealth as they fail to provide adequate identity proofing for patients and healthcare providers. Identity proofing is the process of verifying that the claimed identity of a person matches their actual identity. For healthcare providers, it is essential to verify their practice licenses before allowing them to authenticate themselves to other entities in the network. The verification can only be done by the regulatory authorities who have issued the practice license. At present, remote healthcare applications rely on a centralized IdM where subscribers are forced to trust their service providers in the authentication of other participants. Researchers who have utilized blockchain to provide a decentralized health record system [21,22,23,24,25] also rely on the centralized IdM of hospitals. Therefore, it remains essential to provide a decentralized IdM that allows patients and healthcare providers to authenticate and validate each other in a trusted and reliable manner.

We propose a blockchain-based decentralized identity management for remote healthcare. Health-ID harnesses the power of blockchain technology to safeguard patient information and help ensure regulatory compliance. For that, we invest the automated nature of smart contracts and transparency of the blockchain to provide an IdM with capabilities such as automated provisioning and de-provisioning, user-centric identity governance across domain boundaries, and robust audit and reporting. In this paper, we have three significant contributions that are as follows:

- The architecture of Health-ID is presented, which consists of four actors, namely user, healthcare regulator, blockchain, and cloud storage. The owner can control their own identity by using web tokens for identity attributes. The healthcare regulators provide their attestation after conducting identity proofing. In order to maintain data integrity and auditability, the hash of the identity attribute is uploaded on the blockchain.
- Two smart contracts Health_SC and Registry_SC are deployed to facilitate the authentication and identification process. The health_SC allows users to manage their identity, whereas the registry_SC allows regulators to store the attestations.
- A consortium blockchain is used to implement healthID. The blockchain’s performance effectiveness and computational efficiency are computed using transaction gas cost, transaction per second, number of blocks lost, and block propagation time.

## 2. Related Work

Identity management is described as a set of policies and technologies to control entities’ identities and ensure that the right entities are authorized to utilize relevant resources. The IdM is broadly categorized into centralized, federated, user-centric, and decentralized. In centralized IdM, the identity provider has complete control to manage the identity of users and provide them authentication services. Most of the current services and applications use centralized IdM to create a silo of users where users identified in a specific domain cannot authenticate themselves to other domains [6]. Federated IdM, on the other hand, is an arrangement between two or more organizations to allow users from one domain to authenticate and access services of other domains [26], for instance, single-sign-on systems such as Facebook connect [27]. However, current centralized and federated digital identity models do not allow for complete independence or control by the user. This leads to a privacy issue as users do not know where their data are being utilized. On the other hand, a user-centric identity tries to give control of identity to its owners while reducing identity information disclosure of personal information [27].

The recent emergence of blockchain technology has allowed the development of decentralized IdM. Decentralized IdM will enable consumers to manage and maintain their identity on a blockchain that is not controlled by a single central authority. This allows users to decide what, when, and with whom they want to share their information. Recently, there have been various proposed solutions based on blockchain technology. The decentralized IdM can be broadly categorized into self-sovereign identity and decentralized trusted identity. Self-sovereign identity is a type of user-centric model that requires no central authority, which may lead to the possibility of identity disclosure. In this approach, a unique identifier is used to represent an entity, whereas the attributes of user identity are stored on the blockchain [28]. For instance, uPort is built on Ethereum, which allows its users to manage and keep their identity by using a self-sovereign wallet [18]. In addition, an uPort registry smart contract is used to store the identifiers and their identity attributes. SelfKey [29] is also built on top of the Ethereum blockchain and uses a claim protocol to share identity information with third parties. Sovrin [19] is a decentralized IdM solution that uses a permissioned blockchain network Hyperledger Indy. In Sovrin, only trusted authorities, such as governments, universities, or banks, manage the blockchain by running the consensus protocol [17]. It uses virtual chains to pin the state machine on the network. Evernym [30] uses Sovrin and IOTA blockchain to support a self-sovereign trusted identity for enterprises and organizations. Whereas Blockstack [20] provides a decentralized public-key infrastructure using the Bitcoin blockchain. The major limitation of the self-sovereign approach is that the identities are self-asserted. The user provides identity information with no means to verify its authenticity.

In contrast, the decentralized trusted identity supports identity proofing by allowing trusted third parties to provide identity attestation by verifying public credentials, such as a passport, national identity card, and driving license. ShoCard [17] is the most prominent solution based on a decentralized trusted identity approach. It provides multi-factor authentication without the need to use a password or a username. It uses the bitcoin blockchain and a centralized server to exchange identity information between two parties. However, ShoCard has two major limitations. Firstly, it remains centralized as it relies on a ShoCard server that stores identity certification. Without the centralized server, users are not able to authenticate themselves to third parties. Secondly, based on the bitcoin blockchain, the waiting time for a transaction to be mined is very high, which causes delays to the authentication process. None of these solutions are designed and applied to healthcare scenarios for identity authentication and verification purposes to the best of our knowledge. Furthermore, they do not address the challenges and issues of healthcare applications. Therefore, the eHealth applications that support telehealth still depend upon a centralized IdM [31]. This creates silos of users restricting inter-operable identification between different eHealth applications. Our motivation is to investigate the potential of blockchain technology for the use of IdM in remote healthcare applications.

The most prominent adoption of blockchain technology in healthcare is electronic health records (EHR), aiming to resolve data management, security, and interoperability challenges. For instance, BlocHIE, a blockchain-based medical data exchange platform, is proposed in [32] that uses loosely coupled blockchains to store different types of healthcare data. The system provides on-chain verification to ensure security, privacy, and authentication. An attribute-based signature scheme for multiple users in EHR management is presented in [21], whereas BIoTHR is a novel privacy-preserving IoT-based EHR scheme [33]. In [34], a healthcare management framework is suggested for emergency scenarios that use blockchain technology to ensure access control, authentication, and audibility. Blockchain has enabled an efficient method of data authentication in electronic health records. For instance, a blockchain-based key management scheme is proposed in [22] that provides an efficient mechanism for protecting sensitive medical data in the health blockchain. In [23], a blockchain-based cloud-assisted eHealth system is proposed, which aims to avoid outsourced EHR from malicious modification. A two-way authentication scheme developed in [24] allows data sharing between hospitals. Similarly, Ref. [25] provides cross-platform authentication schemes between hospital networks while ensuring security and privacy. However, these blockchain-based EHR systems are assumed to have a decentralized IdM or rely on a centralized IdM of hospitals. None of these solutions provide an implementation of IdM that allows patients and healthcare providers to authenticate and verify each other irrespective of their healthcare application. Therefore, in this paper, we aim to provide an IdM solution for remote healthcare applications. Patients and healthcare providers will remain in control of their identity by managing and storing their identity attributes. The consortium of healthcare regulators will work the blockchain and provide identity attestation by conducting identity proofing of patients and healthcare providers.

## 3. Blockchain Overview

A Blockchain is a replicated database, managed by a consensus mechanism, in a peer-to-peer network of non-trusting parties. Blockchain can be simply defined as a time-stamped series of data records that are managed by a cluster of nodes [35]. Nodes are computers that are connected in a peer-to-peer network and have an identical copy of the data. A new data entry is validated and transmitted to the entire network to maintain the identical copy of the database. The blockchain data structure consists of a chain of blocks. Each block records transactions validated in a particular period and has not yet been recorded in any prior blocks. These blocks are linked to one another through a cryptographic hash such that each subsequent block contains the hash of the previous block (also called parent) header and hence constitutes a chain. The first block, known as the genesis block, has no reference to a previous block since it has no parent block. This linkability is a cryptographic mechanism that maintains data integrity and immutability in the network. A transaction goes through multiple steps in a blockchain network before it ends up validated by the network. Firstly, a user digitally signs and submits its information as a transaction. Secondly, the transaction is broadcasted to the entire network, where each neighboring node conducts validation for the transaction before relaying it to the next node. Thirdly, the transaction is collected and validated by a validator who includes it in a new proposed block. Fourthly, the consensus mechanism determines the validator who has the right to propose his block to the network. Fifthly, once other nodes validate the block, the block will be added to the chain, and the block will be propagated to all nodes to allow these nodes to update their database. Sixthly, after being recorded in the blockchain, the transaction is considered complete and henceforth consumed by its new owner.

Blockchains can be classified into public, private, and consortium blockchain based on their settings. A public blockchain is permissionless if the platform is publicly open for users without permission from any authority. Generally, in a public blockchain, all transactions are visible to the public. Bitcoin is a well-known example of a public blockchain. On the other hand, a private blockchain is not entirely open for the public to use—a centralized authority controls and defines who has the right to join the network. Thus, a private blockchain is considered to be centralized due to the fact because a single authority maintains the network. In addition, data in a private blockchain is prone to tampering. Prevalent examples of private blockchain include Corda, Hyperledger Fabric, and Hyperledger Sawtooth. A consortium blockchain has different organizations involved to manage a shared blockchain [36]. The authority is distributed among different organizations, and thus, the consortium blockchain is considered to have semi-decentralized management and governance. In a consortium blockchain, only a preselected set of nodes participate in the consensus process. A consortium blockchain is best suited for organizational collaboration. In a consortium blockchain, a limited number of trusted participants are required to validate the block. This is what makes the consortium blockchain highly scalable and guarantees high throughput.

**Figure 1:**
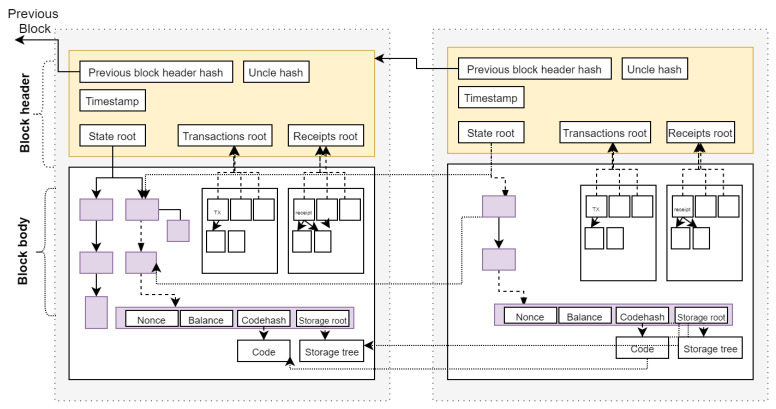
Structure of Ethereum’s chain of blocks.

Ethereum is a stateful blockchain-based computing platform with smart contract functionality that lets users build decentralized applications running on blockchain technology. In addition to the distributed ledger, Ethereum provides a virtual machine, called the Ethereum Virtual Machine (EVM), which can execute scripts written in a high-level programming language (e.g., Solidity). In Ethereum, the blockchain data structure is more complex than in its predecessor, Bitcoin. The block’s header comprises metadata, and its body comprises multiple types of data, namely, transactions, receipts, and system states (account states). Each of these data types is organized into a Merkle tree or a Patricia tree (Radix tree) in the state tree. The state tree (or the account storage tree) is an essential component in the Ethereum ledger. It is used to implement the account model, whereby each account is linked to its related states (account balances, smart contract states, etc.). Any node can parse the tree using the account address and get the updated state without any overhead calculation. The state tree grows each time a change occurs in a state. It grows by adding new nodes (stored in the new block)—holding new states—which points to the nodes (stored in the previous block) containing the old value for the same state (Figure 1). To enforce immutability, Ethereum keeps its root hash in the block header. This tree manages two accounts: the externally owned account (EOA) and the smart contract account. The first type is an account controlled by a private key held by a given user, whereas the second is an account controlled by a smart contract Bytecode. Both accounts are represented by a cryptographically generated address of 20 bytes. To prevent Denial-of-Service (DoS) attack, EVM adopts the gas system, whereby every computation of a program must be paid for upfront in a dedicated unit called gas as defined by the protocol. If the provided amount of gas does not cover the cost of execution, the transaction fails. The block size is controlled by the gas-limit, which the miners define, and a constant rise of the gas-limit happens.

## 4. Remote Healthcare Identity Management System

In this section, we present an IdM solution for remote healthcare services using a consortium Ethereum blockchain. The consortium is managed by healthcare regulators, whereas patients and healthcare providers are the consumers of the IdM identified by a unique health identifier (healthID). Firstly, we define the actors involved and our proposed architecture. Secondly, we discuss the two smart contracts and their functions required for creating, using, and validating healthID. Lastly, we demonstrate the workflow for the healthID registration and authentication process.

### 4.1. Actors

The remote healthcare IdM framework involves five entities. Each entity is briefly described as follows:

1. **User:** A user is the owner of the identity. A user in healthID can be a patient or a healthcare provider. The patient is a consumer of healthcare services, such as diagnosis, treatment, and therapy, whereas a healthcare provider is a professional that is licensed by regulatory authorities to provide healthcare services. Healthcare providers include doctors, nurses, pharmacists, dentists, opticians, midwives, psychologists, and psychiatrists, etc.
2. **Healthcare Regulator:** A regulator is responsible for registering and administrating healthcare providers. Examples of healthcare regulators include the department of health and social care professionals, nursing council, and midwife council, pharmaceutical council, optical council. They are responsible for registering, renewing, and revoking the license of healthcare providers of their respective fields. On the other hand, public hospitals can register patients by verifying their public identity.
3. **Blockchain:** A consortium blockchain is utilized to provide a secure and distributed identity management service for healthcare. The blockchain platform should support smart contracts such as Etherum and Hyperledeger. A piece of code known as the smart contract is deployed over Ethereum to ensure identity management. Two types of smart contracts are deployed, namely Health SC and Registry SC. The healthID of patients and providers is the address of their deployed smart contract over the Ethereum blockchain.
4. **Cloud storage:** The cloud storage system is used to store the identity attributes of patients and healthcare providers. The identity attributes are stored in a JSON object and attested by a regulatory authority to create a JSON Web Token (JWT). The hash of the identity attribute can be used to locate and download a particular identity attribute. The identity owner may select a centralized storage system (such as dropbox) or a distributed cloud storage (such as IPFS) to store their identity attributes.

### 4.2. Proposed Architecture

**Figure 2:**
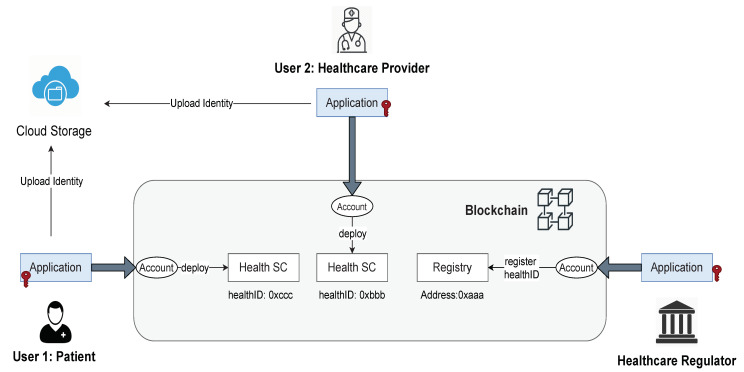
Architecture of the smart healthcare identity management system.

The overview of the remote healthcare IdM is illustrated in Figure 2. The patient, regulator, and provider use their applications to register to the blockchain. The application contains a secure inbuilt wallet having public and private key pairs. The application stores the private key in the secure enclave of the user device that can be utilized by biometric or password authentication. The private key is used to sign attestations and transactions sent to the blockchain. The public key is used to generate an account on the blockchain. The account is further used to deploy smart contracts over the blockchain. Each patient and healthcare provider deploys their smart contract. The healthID is the address of the smart contract deployed by each entity. The unique healthID is used for the identification and authentication process. Healthcare regulators register it by performing off-block identity proofing. The identity proofing of healthcare providers is conducted using their practice license, whereas patients can prove their identity using public identification such as passports, national identity cards, and driving licenses. For instance, the pharmaceutical council will be able to register the healthID of a pharmacist by verifying their practice license. In contrast, a public hospital will be able to register the healthID of a patient by verifying their public identity document. A consortium of healthcare regulators will manage the blockchain. Each member of the consortium will manage a node of the blockchain. When the blockchain is initialized, a specific predefined authority node would be used to validate new blocks on the network. New authority nodes can be included at any time based on the majority decision of existing authority nodes.

**Figure 3:**
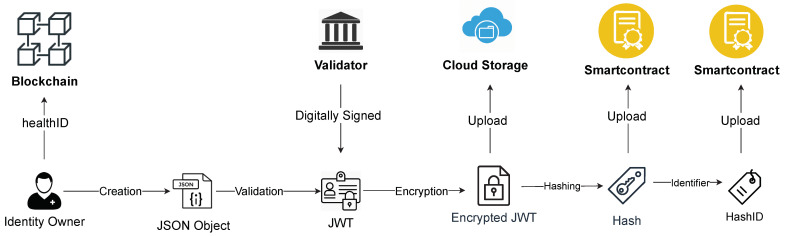
An overview of digital health identity.

In our proposed architecture, the owner can control their own identity. Figure 3 shows how the owner of healthID creates, stores, and manages the identity. The identity is a set of identity attributes describing the owner. We use a JSON (https://www.json.org/json-en.html (accessed on 1 May 2021)) object to define identity attributes, for instance, name, profile picture, license number, and public citizenship number, etc. The JSON attributes are digitally signed by a regulator to create a JSON Web Token (JWT). A JWT (https://jwt.io/ (accessed on 1 May 2021)), which is an open standard to securely transmitting information as a JSON object. The JWT identity token is an attested identity attribute of the owner. It can be used as proof that a particular regulator attests to the claim about the identity of a specific patient or healthcare provider. Attestation can also be a self-signed JSON token. The owner uploads the encrypted JWT identity attributes over a cloud service (Dropbox, IPFS). The hash of the identity attribute is used to ensure the integrity of the data. Each hash is identified by its hashID, which is a unique random number assigned to a particular hash. The hash and hashID are uploaded over the blockchain using the owner’s smart contract.

An example of the JWT having an identity attribute of a doctor’s practice license is provided. The encoded and decoded JWT token is shown below:   

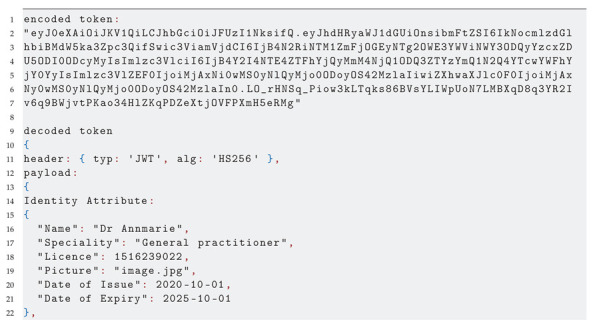

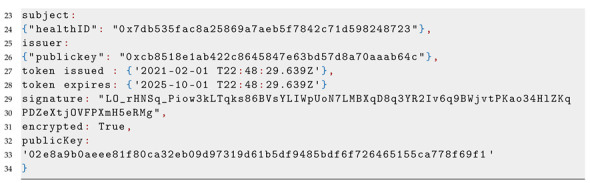





The encoded JWT consists of three parts separated by dots (xxx.yyy.zzz): header, payload, and signature. The header consists of the token type and the signing algorithm such as HMAC, SHA256, or RSA. The payload contains the data about the entity. The signature is created by using an encryption algorithm over the encoded header and payload. In the above example, the JWT is created using the HMAC SHA256 algorithm to create the signature. The payload consists of the license attribute, subject healthID, issuer public key, token issue date, and token expiry date.

### 4.3. Smart Contract

The healthcare IdM consists of two types of smart contracts (Health SC and Registry SC). The Health SC consists of five functions, including set_public_key, retrieve_public_key, set_hash, retrieve_hash and pause_SC, whereas the registry SC consists of functions, register_regulator, verify_regulator, register_attestation, verify_attestation, and revoke_attestation. The algorithm of each function is provided in Algorithms 1–10, respectively. The complete code of the smart contract is available in the github repository (https://github.com/ibrahimtariqjaved/healthid (accessed on 1 May 2021)).

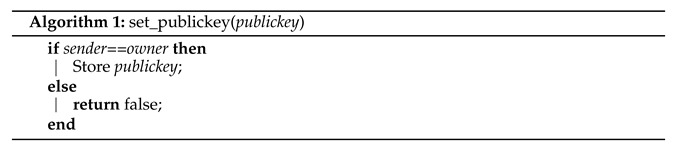

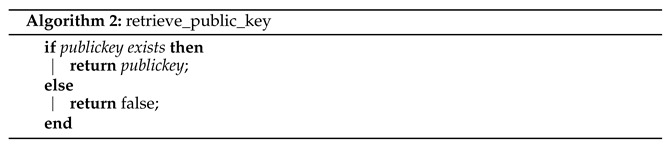

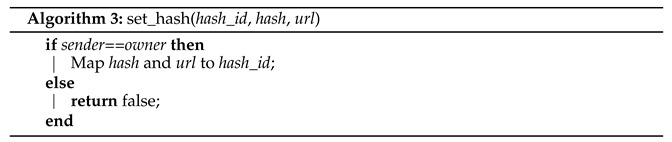

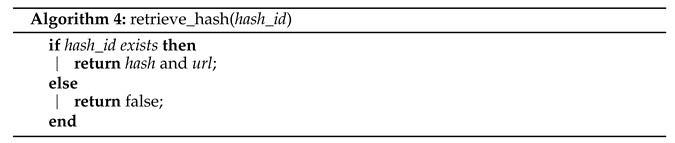

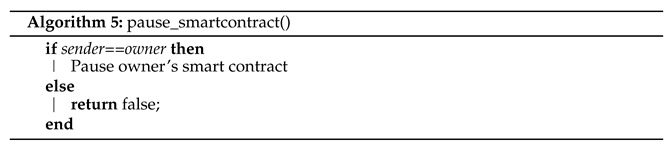

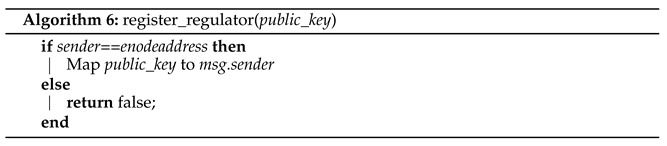

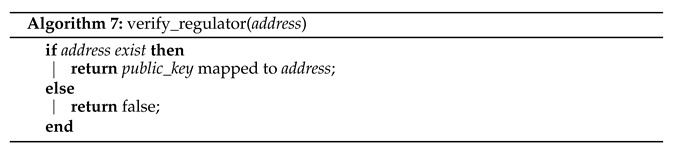

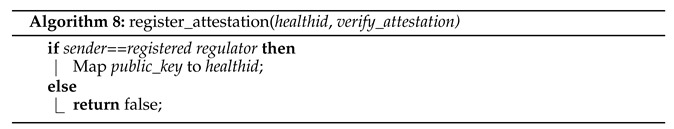

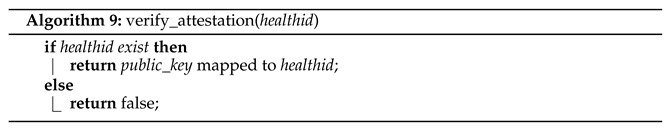

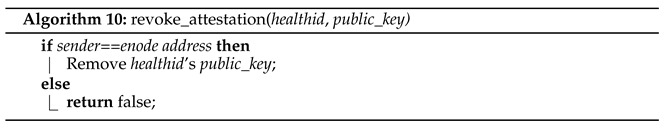





















The description of each function is provided as follows:

1. *set_public_key:* This function allows the owner of the smart contract to upload and store its public key over the Ethereum network. Only the owner of the smart contract will be allowed to upload their public key.
2. *retrieve_public_key:* This function allows anyone to retrieve the public key of the owner by using their healthID. Retrieving the public key from this function ensures that the public key belongs to the entity having the healthID.
3. *set_hash:* This function allows the owner to store the hash and hashID of their identity attribute. The function stores the hash with the corresponding hashID of the identity attribute. Only the owner of the smart contract is allowed to upload the hash.
4. *retrieve_hash:* This function is used to extract the hash by using the hashID of the identity attribute required. The function uses the hashID to locate and return the corresponding hash of the required identity attribute.
5. *pause_SC:* This function allows the owner of the smart contract to pause and unpause the contract. If the smart contract is paused, no one will access the hash of the identity attribute.
6. *register_regulator:* This function allows regulators to register themselves and upload their public key. The public key is stored with their corresponding Ethereum address. This function will only register the regulator who is operating as a node of the blockchain.
7. *verify_regulator:* This function allows anyone to retrieve the public key of regulators using their Ethereum address. In addition, this function ensures that the regulator having a particular Ethereum address is registered on the network.
8. *register_attestation:* This function allows regulators to register the healthID and public key of patients and healthcare providers. The Ethereum address of the regulator is also stored with it. Only the registered regulators will be able to use this function.
9. *verify_attestation:* This function allows anyone to extract the public key of the registered healthID. In addition, the function returns the public key and Ethereum address of the regulator who provided the attestation.
10. *revoke_attestation:* This function allows the regulator to revoke the attestation of a particular patient or healthcare provider using their healthID.

### 4.4. Identification and Authentication Workflow

**Figure 4:**
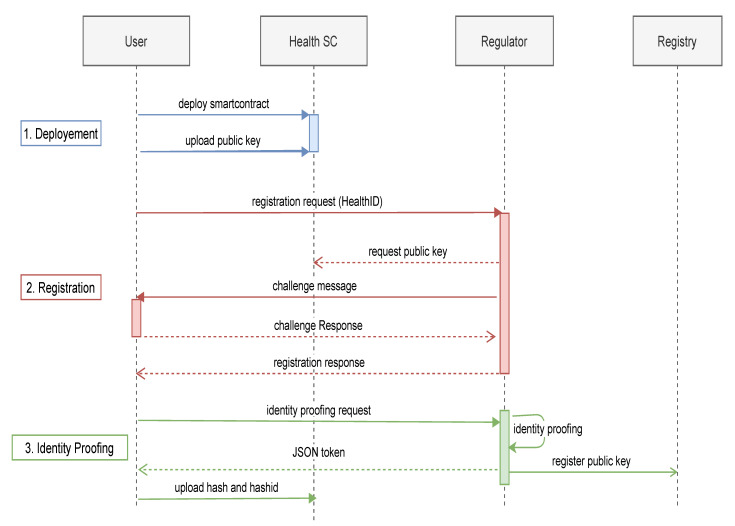
Identification sequence diagram.

Identity management consists of the identification and authentication process. In identification, the patients and providers would register and identify themselves to the system using their applications. In the authentication step, the identity owner would prove their identity to a third party using their validated healthID and identity token. The system supports single-sign-on, in which the individual, once logged into its application, would be able to authenticate itself to different entities. The workflow of each process is described using a sequence diagram. The process of identification is presented in Figure 4. The identification process of patients and healthcare providers is the same. In the Figure, we take the example of a healthcare provider registering to the remote healthcare IdM by following the identification process. The identification process consists of the followings steps:

1. Deployment: In the first step, the healthcare provider will use the EoA account to deploy a smart contract on the Ethereum blockchain using their application. The address of the smart contract would be the digital healthID of the healthcare provider.
2. Registration: In the registration step, a request along with the owner’s healthID is sent to the regulator by the provider. The regulator will use the healthID to request the public key from the provider’s health SC using *retrieve_publickey* function. Using the public key, the regulator will encrypt a challenge message and send it to the provider. If the provider decrypts using its private key and responds successfully, this ensures that the provider is the real owner of the healthID and public key.
3. Identity proofing: In this step, the provider would be required to prove their identity by presenting their practice license. The provider may be required to physically or remotely present their license based on the policy of the healthcare regulator. After proofing is conducted successfully, the regulator registers the healthID and public key of the provider in the registry SC using the *register_attestation* function. The regulator further signs the identity attribute and provides an identity token (JWT) to the provider.

**Figure 5:**
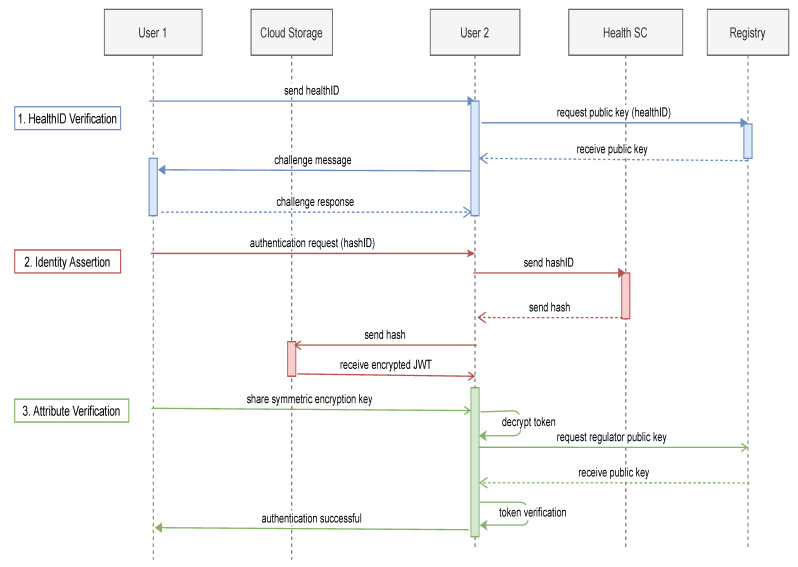
Authentication sequence diagram.

After identification, the healthID is registered, and the owner receives a signed identity token. The provider stores the identity token on its device or uploads it to the cloud. The hashID and hash of the identity token are uploaded to the provider’s Health SC using *set_hash*. Now the healthcare provider can authenticate themself to anyone before providing their healthcare services. The process of authentication is presented in Figure 5. The authentication process for a provider and a patient is similar. We present how a provider authenticates itself to a patient. The steps for the authentication process are discussed below:

1. HealthID Verification: In the first step, the healthcare provider sends the healthID to the patient. The patient uses the healthID to extract the public key from registry SC using the *verify_attestation* function. This ensures that the attested healthID is verified and registered by a particular regulator. The Ethereum address of the regulator providing the attestation is also provided. Next, the provider’s public key is used to send a challenge message to the healthcare provider. If the response is correct, this guarantees that the public key and healthID belong to the healthcare provider.
2. Identity Assertion: In this step, identity assertion is used by the patient to authenticate the provider. The provider sends the hashID of the required identity token. The patient uses the hashID to receive the corresponding hash from the provider’s Health SC using the *retrieve_hash* function. This allows the blockchain to keep a record of each authentication taking place. The patient then uses the hash to retrieve the identity token (JWT) from cloud storage.
3. Attribute Verification: The provider shares the symmetric key securely, which is used to decrypt the identity token received from the cloud. To verify the signature of the regulator, the public key of the regulator is requested using its Ethereum address from the *verify_regulator* function of Registry SC. This allows the patient to verify that the regulator is registered. The public key received is used to verify the identity token. This proves that the identity assertion is validated and attested by the regulator.

### 4.5. Discussion

The healthcare solutions presented in [21,22,23,24,25,32,33,34] only focus on decentralized healthcare record management systems. These solutions either assume to have a decentralized IdM or rely on a centralized IdM of hospitals. None of these solutions provide a detailed implementation of an IdM system where patients and healthcare providers can authenticate and verify each other irrespective of their healthcare application. On the other hand, the existing blockchain-based IdM solutions, such as [17,18,19,20,29,30], provide a single sign-on authentication system where users have self-asserted identities by creating their profiles. These systems lack adequate identity proofing to validate the identity information. It is essential to conduct identity proofing in healthcare applications before allowing patients and healthcare providers to authenticate each other. In the healthcare sector, healthcare providers are licensed by regulatory authorities to provide healthcare services. For instance, a doctor would be certified by healthcare professionals, whereas the pharmaceutical council would approve a pharmacist. Therefore, the identity attestation should be conducted by the relevant regulatory authority. Compared to the existing solutions, the healthID facilitates patients and healthcare providers to authenticate each other without depending on their eHealth provider. HealthID harnesses the power of blockchain technology to safeguard patient information and help ensure regulatory compliance by allowing identity proofing. The complete implementation of the solution is presented in the next section.

## 5. Implementation and Evaluation

In this section, we implement the smart healthcare identity framework on a consortium Ethereum network. We explain the setup that is used to deploy a smart contract over the blockchain. We further present and discuss the performance of deployed consortium blockchain.

### 5.1. Blockchain Deployment

We choose a consortium blockchain to implement healthID. Consortium removes the centralized control as a group of healthcare regulators manages it in contrast to a single entity in a private blockchain. Furthermore, it is more privacy-ensuring and efficient due to the smaller node count compared to the public blockchain. We deployed the consortium blockchain using the Ethereum network on five nodes. Each node is set up on an EC2 virtual machine using an AWS cloud service. The virtual machine has 1 GB RAM and 20 GB storage with a Linux Ubuntu operating system. Each virtual machine is configured with the Go Ethereum client (Geth (https://github.com/ethereum/go-ethereum/wiki/geth (accessed on 1 May 2021))) that is implemented in Go language. The five nodes running the Geth client are connected using their eNode addresses. After initializing the blockchain, we used Remix IDE (https://remix.ethereum.org/ (accessed on 1 May 2021)) to compile healthID smart contracts implemented in solidity language. We further used the Metamask (https://metamask.io (accessed on 1 May 2021)) wallet to deploy the smart contract onto our consortium blockchain. After deploying our smart contacts, we submit dummy transactions over the blockchain. The commands used to set up and initialize the Ethereum blockchain are provided in the GitHub repository (https://github.com/ibrahimtariqjaved/healthid (accessed on 1 May 2021)).

The PoA consensus agreement is used to deploy the consortium blockchain. The PoA blockchain in Ethereum is named the Clique network. The nodes that validate the block are called sealers. In PoA, the network consensus is achieved by a majority agreement among the sealer nodes. The genesis JSON file configures the network and initializes the first block on the blockchain network. A sample JSON genesis file is presented in Listing 1. The genesis file contains several parameters that are essential to configure the PoA consensus. The *chainID* is used to allocate an identifier to the network. The *parentHash* parameter defines the hash of the previous block, which is set to 0 as the genesis block has no parent block. The *gasLimit* is used to set the limit of gas that can be used per block. Gas refers to the cost required to perform a transaction on the network. The *gasLimit* defines how many transactions can be part of each block. The *Epoch Period* defines the size of each block as it is the time set between two successive blocks. The block-time is used to determine the size of each block. The *extraData* is used to set the addresses of sealers when initializing the blockchain. Sealers are nodes that can validate and include a transaction on a block. In our case, healthcare regulators are set as sealers of the network. A consortium is deployed by initializing a set of sealers in the genesis block. The majority voting of existing sealers can include a new sealer. The genesis file is used to set the initial parameters necessary for the blockchain. We analyze the performance of blockchain by using three parameters (i) Block-Time, (ii) Gas-Limit, and (iii) Sealers Number. The consortium blockchain is tested by varying these three parameters. For each configuration, we run the blockchain for one hour to compute the performance.

**Listing 1:**
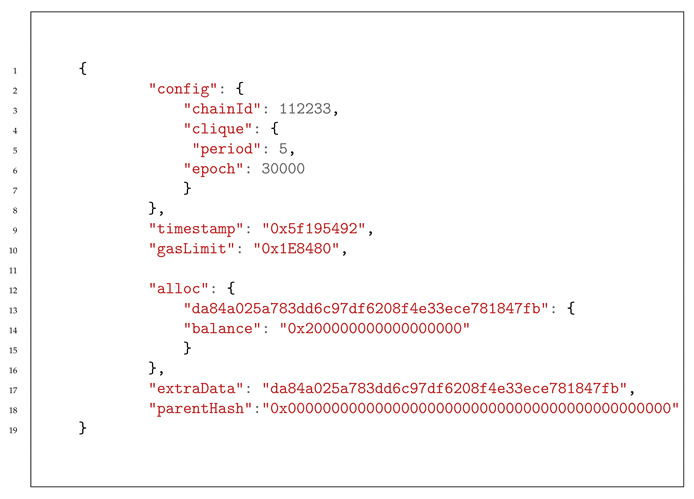
JSON genesis.

### 5.2. Performance Evaluation

We compute the performance of the healthcare identity framework on a consortium blockchain using the Ethereum network. We use four metrics to compute the performance of the Ethereum blockchain:

1. *Transaction gas cost (TGS):* TGS is the amount of gas needed to run a smart contract transaction on the Ethereum blockchain network. It represents the efficiency of the smart contract in terms of its execution. *TGS* needs to be minimized to achieve higher efficiency and lower delays in the network as each transaction is executed over all nodes of the blockchain.
2. *Transaction Per Second (TPS):* TPS is the total transactions that can be carried out on the blockchain in one second. It is computed using the *gasLimit* divided by the TGS and $Block-time_{measured}$. Where The $Block-time_{measured}$ is the actual block-time recorded from the geth console. The $Block-time_{measured}$ may differ from the block-time set in genesis due to synchronization and network delays.
3. *Number of Blocks Lost (NBL):* The *NBL* is the number of blocks lost in the network. The NBL can be measured directly from the geth console. A block is lost when the sealer delays broadcasting the signed block for a specific time. After that, the block is replaced by a new block proposed by a backup sealer. The block that was required to be added to the blockchain is considered lost. Therefore, the number of blocks lost produces lag in the blockchain network. To reduce the delay of block generation, the *NBL* needs to be minimized.
4. *Block Propagation Time (BPT):* BPT is the time that is needed for a new block to be distributed to the majority set of nodes present in the network. Each block is propagated to all nodes in the network after validation using a defined broadcast protocol. Thus, for a block to reach the entire network, it passes through approximately seven intermediary nodes. The propagation time for each block can be extracted from the geth console.

**Figure 6:**
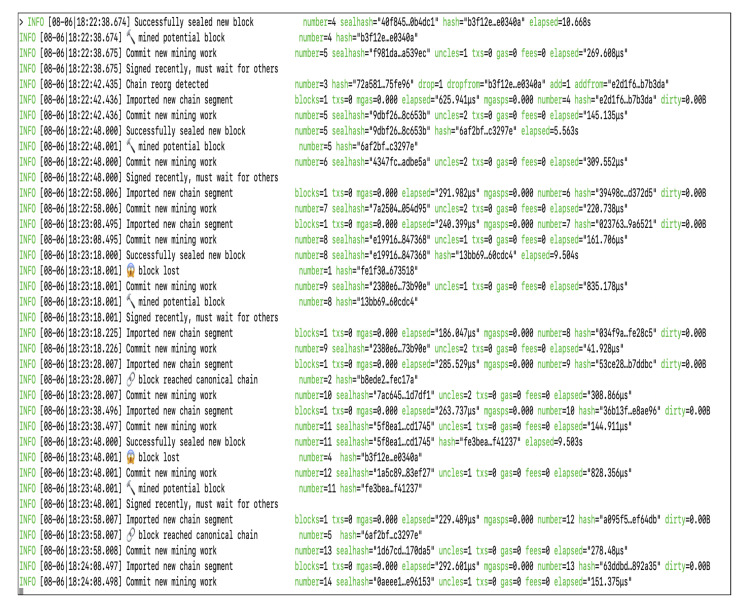
Screenshot of geth console.

In order to compute the performance, we ran the blockchain for one hour for each setting. The information regarding each block is extracted from the geth console. The screenshot of the geth console is shown in Figure 6. The $Block-time_{measured}$, NBL and BPT are directly extracted from the geth console.

#### 5.2.1. Transaction Gas Cost of HealthID Smart Contract

In this subsection, we compute the performance of our smart contracts in terms of their complexity. We compute the TGS for deploying smart contracts and their functions, as presented in Table 1. However, PoA does not require any cost to be paid. The TGS is a good metric to check the complexity of the smart contract. The higher the gas cost, the more time it will take to execute the function on the blockchain. The TGS for deploying Health SC requires 485561 gas, as shown in Table 1 (a). The deployment cost of the registry smart contract is almost similar, as seen in Table 1 (b). This cost is required only once upon deployment of the smart contract. Regarding the smart contract functions, we can observe that none of the functions has TGS above 50,000 gas. The TGS of *set_publickey* and *set_hash*, *register_ regulator* and *register_attestation* functions are above 40,000 gas as they require mapping of 32 bytes addresses. From Section 4.4, we can observe that for the identification process, five smart contract functions are required, namely *Patient_SC deployment*, *set_publickey*, *get_publickey*, *register_attestation* and *set_hash*. Therefore, for the identification process, a total of 485,561 + 44,538 + 22,351 + 66,327 + 46,887 = 623,465 gas is required. The cost of identification is high due to smart contract deployment. However, this cost is required only once for initializing the smart contract on the blockchain. After the smart contract is deployed, the process of identity proofing will only require a *TGS* of 135,565 gas. On the other hand, the authentication process requires only three functions *verify_attestation*, *get_hash* and *verify_regulator*. For which a *TGS* of 24,911 + 22,351 + 23,920 = 71,182 gas is required. This shows that the authentication process requires very little computational power and can be executed quickly.

#### 5.2.2. Effect of Block-Time and Gas-Limit on Blockchain Performance

**Figure 7:**
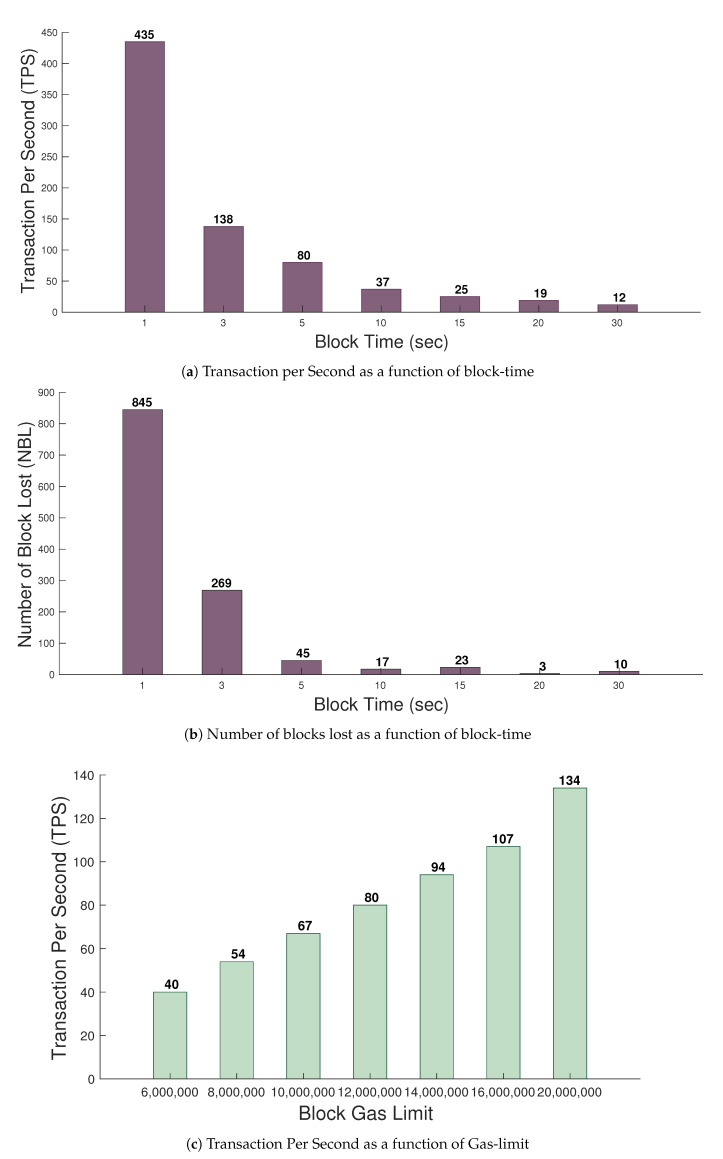
Effect of block-time and Gas-limit on blockchain performance.

In this subsection, we evaluate the effect of block-time and gas-limit on the performance of the blockchain. To determine the effect of these two parameters on blockchain performance, we compute TPS and NBL. As discussed earlier, *TPS* represents the number of transactions processed by the blockchain in a second, whereas NBL represents the number of blocks lost during block creation. Figure 7a shows *TPS* with respect to block-time. We can observe that *TPS* decreases rapidly when block-time is increased. To achieve a high *TPS*, a small value of block-time is required. However, we further observed that lower values of block-time have a high number of *NBL*, as shown in Figure 7b. The lost blocks introduce delay in the network as new blocks are required to replace them. A large decrease in NBL is observed for block-time between 1 to 5. This is because, at high block-time, sealers have additional time to validate the transactions. Therefore, we suggest a block-time between 5 and 10. For a block-time of 7, *TPS* of around 100 can be achieved with a low number of lost blocks. We further computed the effect of gas-limit on the TPS. From Figure 7c, we can observe the effect of gas-limit on the TPS. For a block-time of 7, we varied the gas-limit from 60,000,000 to 200,000,000. Currently, the public Ethereum blockchain [37] uses a gas-limit of 20,000,000. However, we can use higher gas-limits for a private blockchain as there are a limited number of nodes present in the network. To achieve higher TPS in a private blockchain, we can increase the gas-limit. This will allow a high number of transactions to be accommodated inside the block-time. For a gas-limit of 200,000,000, we observed that TPS reached above 130. We also experimented with the effect of gas-limit on NBL. However, we did not found any effect.

#### 5.2.3. Effect of Sealers on Blockchain Performance

**Figure 8:**
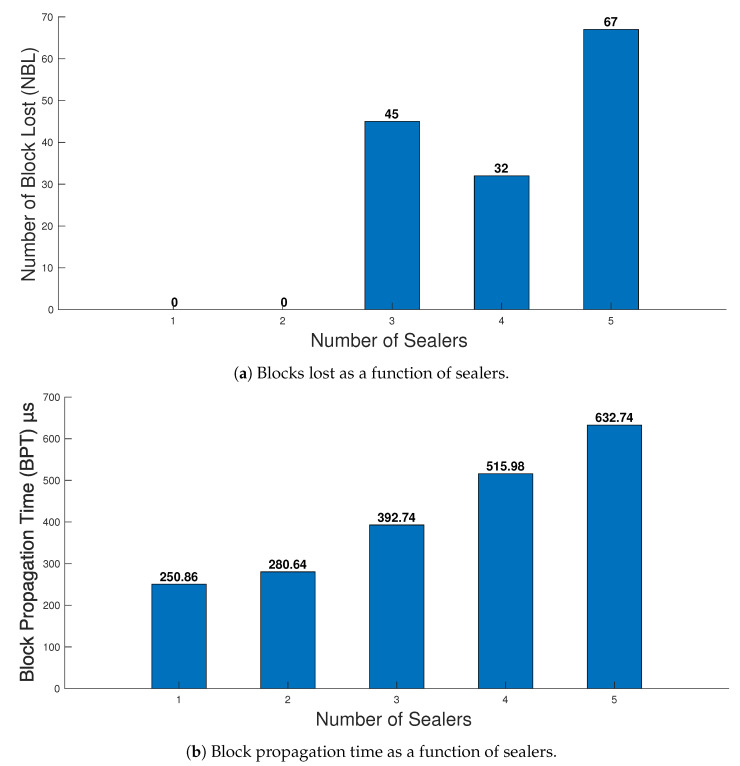
Effect of sealers on blockchain performance.

In this subsection, we observe the effect of sealers on blockchain performance. In the five-node network, we set the number of sealers from one to five and observed their effect on NBL and BPT. First, we tried to observe the NBL in one hour for sealers in the network, as shown in Figure 8a. It can be seen that there were no lost blocks until three sealers were selected. However, when more than three sealers were selected from five nodes, a high number of NBL were observed. The high number of NBL is caused due to synchronization between sealers. In order to validate the block as at least 51%, sealers need to verify it. Therefore if more nodes are made sealers, this may cause NBL to increase. Therefore the sealers should not be more than half the number of nodes present in the network. We further observed the BPT in the network concerning the number of sealers, as shown in Figure 8b. As discussed earlier, BPT is the time required for a block to be distributed to most nodes in the network. It can be observed from the Figure that the BPT is strongly dependent on the number of sealers. More synchronization issues were observed when the number of sealers was added to the network, which was the reason behind the higher propagation delay. As seen from the Figure, increasing the number of sealers to five, the BPT is increased by around 381.88. Therefore, to reduce the network minimum possible number, several sealers should be selected to run the network.

## 6. Conclusions and Future Work

Identity management has gained significant attention in recent years. However, the interoperability, regulation compliance, and security of identity management solutions are still complex challenges yet to be solved. The end-user can only trust the eHealth service provider regarding the safety and protection of its data and attributes. This paper presented a portable and privacy-preserving decentralized identity management solution with an application for remote healthcare services that shifts the control over identity information from service provider to end-user. The proposed system allows patients and healthcare providers to authenticate themselves across different eHealth domains without relying on a central service provider. The healthID approach harnesses a set of smart contracts to tokenize the identity of the network’s entities and end-users, such that a unique healthID identifies each entity. A healthcare regulator digitally signs the identity attributes to create a JSON Web Token, which the owner stores. The identity attributes are further indexed on the blockchain using the owner’s smart contract to ensure immutability and traceability.

To evaluate the performance of the proposed system, we implemented the smart contracts over an Ethereum blockchain managed by a consortium of healthcare regulators and assessed its efficiency in terms of gas cost and speed. We observed that a throughput higher than 100 TPS could be achieved over the consortium blockchain, which is around eight times greater than the throughput of a public blockchain. Moreover, we evaluated the blocks lost in the presence of a different number of sealers. We found out that the number of sealers should be less than half the network number to minimize propagation and synchronization delays for optimized performance. We intend to extend our work to securely manage and store patient’s health records by using zero-knowledge proofs for future work. Our proposed IdM solution would be applied to the real health ecosystem using a cancer patient health record database. We also plan to integrate the blockchain with a distributed storage system, such as IPFS, and measure its performance.

## Figures and Tables

**Table 1:** Gas cost for healthcare identity smart contracts.

**(a) Gas Cost for Health Smart Contract**		
**No**	**Contract Transaction**	**TGS**
1	Deploy Health_SC	485,561
2	set_publickey	44,538
3	get_publickey	22,351
4	set_hash	46,887
5	get_hash	24,434
6	pause_smartcontract	43,436
**(b) Gasgas Cost for Registry Smart Contract**		
**No**	**Contract Transaction**	**TGS**
1	Deploy Registry_SC	474,939
2	register_regulator	43,757
3	verify_regulator	23,920
4	register_attestation	66,327
5	verify_attestation	24,911
6	revoke_attestation	14,492
